# Refuse of admission to intensive care: analysis of a score

**DOI:** 10.1186/2197-425X-3-S1-A333

**Published:** 2015-10-01

**Authors:** M Arlandis Tomas, E Bisbal Andres, R Reig Valero, D Ferrándiz Sellés, L Mateu Campos, A Ortiz Martinez, C Martinez Valero, J Rodriguez Portillo

**Affiliations:** Hospital General de Castellon, Castellón de la Plana, Spain

## Objectives

To analyze whether the parameters used to refuse admission to the ICU have been successful in identifying those patients rejected and its relationship with the final outcome.

## Methods

Prospective, descriptive study in a multi-disciplinary ICU, university referral hospital from June 2013 to May 2014. Demographic data, severity of illness based on sequential organ failure assessment (SOFA), degree of independence (Barthel index) and comorbidities (Charlson Index) were documented. Age was categorized into subgroups, assigning a score of 1-6 upstream ( < 40; 40-60; 61-70; 71-75; 76-80;> 80 years), SOFA score give a possible score of 0 to 24, Barthel Index of 1-5 points and Charlson Index of 1-3 points. Adding the four variables, the final score had a rating from 9 to 41 points.

## Results

One thousand four hundred fifty-eight (1458) patients were assessed for admission in ICU, and 397 (mean age 73 and 63,2% males) were refused. Reasons for refusal were low severity (70.8%), limitation of therapeutic effort (28,2%) and patient or relatives refusal to UCI admission (1%). Global mortality in the group where UCI admission was refused was 19.85%. Hospital mortality in this group was 21.7%, 17.9% related to the cause of refusal and 3.8% was unrelated.

The score for those patients refused due to low severity was 8.9 ± 3.4 and those refused for limitation of therapeutic effort was 14.4 ± 3.3. The score was higher in non-survivors (9.6 ± 3.7) than in survivors (14.2 ± 3.6).

The area under the receiver operating characteristic (ROC) curve between global mortality and score was 0.82 (95% confidence interval (CI): 0.77 to 0.87) where the cutoff between 11 and 12 points was established.

## Conclusions

The parameters used to create a new score allow us in our particular setting identify patients who should not benefit from ICU care.

